# Convenient Benzylic Bromination of 2‑Hydroxy-5-methylisophthalaldehyde

**DOI:** 10.1021/acsomega.5c11342

**Published:** 2026-01-23

**Authors:** Nikita Žoglo, Anton Mastitski, Vladislav Ivanistsev, Nadezda Kongi

**Affiliations:** Institute of Chemistry, 37546 University of Tartu, Ravila 14a, 50411 Tartu, Estonia

## Abstract

A convenient and scalable method for the selective benzylic bromination of 2-hydroxy-5-methylisophthalaldehyde was developed, previously unreported in the literature. Direct Wohl–Ziegler conditions failed, suggesting the investigation of various protection strategies. A combination of methyl ether and geminal diacetate protecting groups gave rise to clean photochemical bromination with *N*-bromosuccinimide in PhCl, affording the benzyl bromide in 39% yield after recrystallization, which can be further functionalized or deprotected. This robust three-step procedure provides a practical pathway for the functionalization of isophthalaldehyde derivatives.

## Graphical abstract

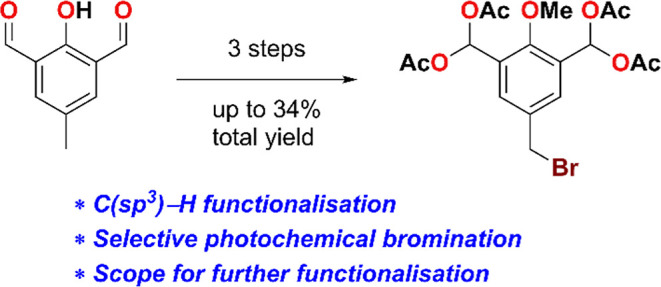

## Introduction

The bromination reaction is a versatile and crucial chemical transformation.^1^ It has long been used in analytical chemistry, e.g., to determine the number of conjugated bonds in olefins,^2^ and has also found widespread use in preparative organic synthesis.^3–5^ The application of bromides ranges from medicinal drugs to flame retardants^6,7^ and other important intermediates in synthetic transformations.^8,9^ Introduction of bromine into the moiety of the molecule could also be utilized for radioactive isotope labeling^10,11^ or for the addition of a good leaving group and its further conversion in the synthetic pathway.^12,13^ Organobromides are used as starting materials in the preparation of various Grignard reagents^14–16^ and organic compounds.^17^ Many organohalides, particularly bromides, are widely used in transition metal-catalyzed cross-coupling reactions, e.g., Suzuki–Miyaura,^18,19^ Heck,^20,21^ Negishi,^22,23^ and Sonogashira^24,25^ coupling. Thus, bromination is a standard, straightforward, and routine procedure in organic preparative chemistry. It is usually achieved through either an electrophilic or a radical process. Electrophilic bromination of aromatic compounds could be performed through a Lewis acid-catalyzed reaction with elemental bromine,^26,27^ while unsaturated hydrocarbons will react with bromine without any catalysts.^28^ In the presence of acid, i.e., acetic acid, bromine reacts with ketones to produce a C–Br bond at the “alpha” position.^29^ As for the radical process, the most standard procedure is performed using a *N*-bromosuccinimide (NBS) brominating agent, in CCl_4_ solution with benzoyl peroxide (BzO)_2_ as a radical initiator, e.g., Wohl–Ziegler bromination.^30,31^ Azobis­(isobutyronitrile) (AIBN) could also be used instead of benzoyl peroxide,^31^ or, alternatively, the initiation could be achieved through photochemical excitation.^32^

For one of the ongoing projects in our research group, we were required to install a bromide group on the benzylic position of 2-hydroxy-5-methylisophthalaldehyde (**1**) to obtain compound **1b** or its derivatives (Scheme 1). However, the initial literature search suggested that there were seemingly no clear synthetic procedures for the synthesis of compound **1b** or its protected analogues. Only a few chemical providers can offer similar brominated products, but they require on-demand synthesis and have quite extortionate prices. Armed with this information, we decided to investigate whether it would be possible to introduce the bromine atom into the benzylic position starting from compound **1**, which is a readily available and relatively inexpensive starting material.

**Scheme 1:**
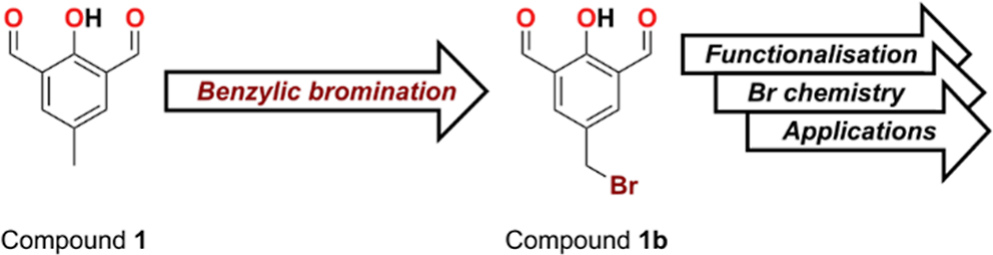
Initial Synthetic Strategy

## Results and Discussion

In our experiments, we explored different reaction conditions for the radical bromination of the methyl (Me) group in compound **1** using NBS. Two different solvent systems were tested: chlorobenzene (PhCl) at 100 °C and CCl_4_ at reflux. The radical process was activated using either benzoyl peroxide (BzO)_2_ or through a photochemical process by irradiation with a 400 W floodlight. The effect of varying protecting groups on the hydroxyl and formyl functional groups was also studied. All of our findings are summarized in Table 1.

**Table 1:** Bromination of Compound **1** Derivatives

entry	phenol protection	formyl protection	solvent, temperature	activator, equiv	bromination reagent, equiv	yield	comments
**A**	–	–	PhCl, 100 °C	(BzO)_2_, 0.1	NBS, 1.2	–	no bromination
**B**	–	–	CCl_4_, reflux	(BzO)_2_, 0.2	NBS, 1.1	–	no bromination
**C**	Boc	–	PhCl, 100 °C	(BzO)_2_, 0.1	NBS, 1.2	–	no bromination
**D**	Me	–	CCl_4_, reflux	(BzO)_2_, 0.2	NBS, 1.1	–	no bromination
**E**	Ac	(OAc)_2_	CCl_4_, reflux	(BzO)_2_, 0.2	NBS, 1.1	(not purified further)	traces of benzylic + aromatic bromination
**F**	Me	(OAc)_2_	CCl_4_, reflux	(BzO)_2_, 0.1	NBS, 1.2	(not purified further)	benzylic + aromatic bromination
**G**	Me	(OAc)_2_	CCl_4_, reflux	*hv* ^a^	NBS, 1.2	70%	benzylic bromination observed + easily purified
**H**	Me	(OAc)_2_	PhCl, 120 °C	*hv* ^a^	NBS, 1.2	(not purified further)	benzylic mono- and dibromination
**I**	Me	(OAc)_2_	PhCl, 80 °C	*hv* ^a^	NBS, 1.2	39% (after second EA/hexane recryst.)	benzylic mono- and dibromination
**J**	Me	(OAc)_2_	PhCl, 80 °C	*hv* ^a^	NBS, 1.4	(not purified further)	benzylic mono- and dibromination

a A 400 W floodlight was used as a source of radiation.

In our initial experiments, we tried direct radical bromination of compound **1**, without any protecting groups on the hydroxyl or formyl groups. First, radical bromination using 1.1 to 1.2 equiv of NBS, in the presence of 0.1 to 0.2 equiv of (BzO)_2_, was investigated in PhCl at 100 °C and in CCl_4_ at reflux (Table 1, entries **A** and **B**). Both tests yielded a mixture of various side products with no observable signs of benzylic bromination, hinting at the fact that unprotected hydroxyl and formyl groups might be hindering the process of radical bromination by either destroying the benzoyl peroxide, terminating the radical chain reaction, or undergoing oxidation during the reaction. This was confirmed while examining the bromination of the compound **1** derivatives with only the hydroxyl group protected, with either a *tert*-butyloxycarbonyl (Boc) ester (Table 1, entry **C**) or methyl ether protection (Table 1, entry **D**). Boc protection was installed by a reaction of **1** with a stoichiometric amount of Boc_2_O in the presence of DIPEA base in DCM, while methyl ether protection was achieved by the reaction of **1** with methyl iodide (MeI) in the presence of K_2_CO_3_ in DMF. Protection of just the hydroxyl group yielded no success at the desired benzylic bromination, producing a messy mixture of side products (potentially some degree of aromatic core bromination) and unreacted starting material. In some tests, we also observed Boc group cleavage at an elevated temperature.

Based on these findings, we decided to investigate the acylal protection of the formyl groups due to the very mild reaction conditions required for their synthesis. In the next test, compound **1** was set to react with an excess of acetic anhydride (Ac_2_O) under solvent-free conditions in the presence of a catalytic amount of methanesulfonic acid (MsOH). This provided a fully protected derivative of **1** with the hydroxyl converted into the acetate ester and the formyl groups transformed into 1,1-diacetate moieties. Subsequent radical bromination (Table 1, entry **E**), using the aforementioned nonphotochemical reaction conditions, finally gave rise to trace amounts of benzylic bromination, observed by the shift in the NMR signal of the Me group, consistent with the presence of an adjacent electronegative Br atom. However, the conversion and purity of the reaction were far from perfect, with side products of aromatic bromination still present in the mixture. We also suspected that the steric bulk of numerous acetate groups in the acylal greatly hinders the acetate ester on the hydroxyl, perhaps making it more susceptible to undergo deprotection, as observed with the Boc group.

Going forward, we decided to use a more robust, stable, and less sterically hindering methyl protection on the hydroxyl, while formyl groups were converted into 1,1-diacetates (compound **3**, Scheme 2).

**Scheme 2:**
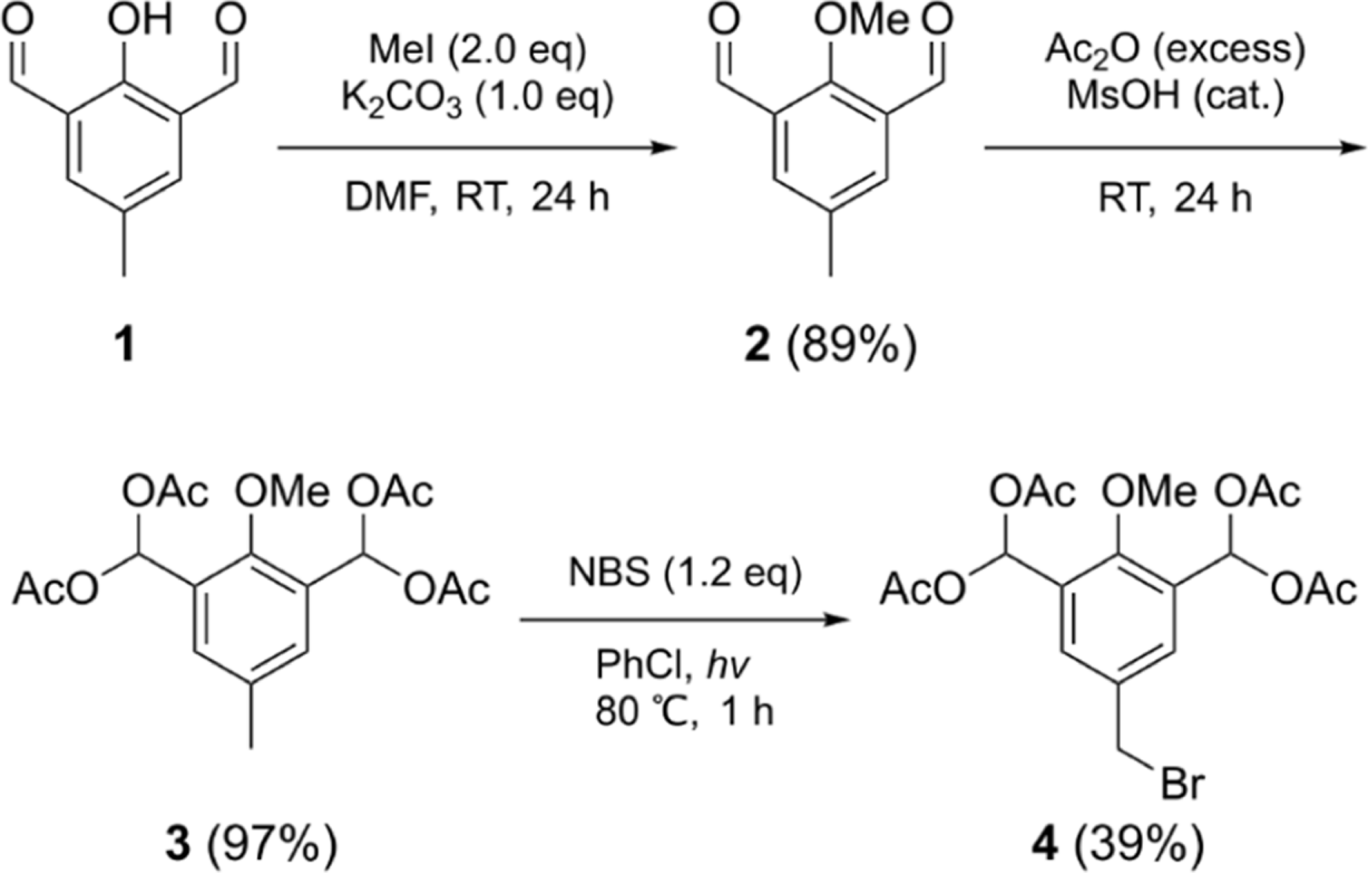
Three-Step Synthesis of (5-(Bromomethyl)-2-methoxy-1,3-phenylene)­bis­(methanetriyl) tetraacetate (**4**)

On their own, these protecting groups had shown promising results in terms of bromination. However, the combination of these two protecting groups gave the best result in all of our experiments. A greater proportion of the benzyl bromide was observed with the previously mentioned reaction conditions of NBS and (BzO)_2_ in CCl_4_ (Table 1, entry **F**), while some side products were also present in the crude mixture. To improve conversion, we decided to switch from activation by benzoyl peroxide to a photochemical bromination setup, which has given rise to quite remarkable results in the literature.^33^ Instead of the radical initiator, the reaction mixture in CCl_4_ was irradiated with a 400 W floodlight under reflux. This new setup produced a mixture of the unreacted starting material and the desired benzyl bromide (compound **4**, Scheme 2) with good relative conversion and no other side products (Table 1, entry **G**). The crude mixture was easily purified by recrystallization to separate out the bromide. Although CCl_4_ gave the best results, due to its high toxicity, carcinogenic nature, and poor environmental impact, we decided to explore a “greener” alternative, namely, chlorobenzene (PhCl), previously reported in the literature.^33^ In contrast to CCl_4_, chlorobenzene has lower volatility, a higher boiling point, lower toxicity, and fewer environmental hazards.

The first test (Table 1, entry **H**) was performed at 120 °C, with all other conditions remaining the same. This produced a mixture of the desired monobrominated product, some dibrominated product, and unreacted starting material. The following test (Table 1, entry **I**) was performed at a slightly lower temperature of 80 °C, mimicking the CCl_4_ refluxing conditions, to see the effect on the bromination reaction conversion. Slightly milder heating was found to give the same conversion of around 75% benzyl monobromide in the crude mixture. We also attempted to increase the NBS loading (Table 1, entry **J**), which ultimately resulted in a worse relative conversion and more side products. Taking all of this information into account, we decided to proceed with the reaction conditions described for entry **I**. The crude mixture of the monobrominated and dibrominated compound and starting material was recrystallized from ethyl acetate by the addition of hexane. Compound **4** was obtained in 47% yield after the first recrystallization; however, slight traces of the dibrominated impurity were still present. It should be noted that at this point, the obtained compound **4** is pure enough for the majority of synthetic applications; however, recrystallization was performed a second time to isolate the target compound in 39% yield. The obtained benzyl bromide could then be used in further synthetic steps, e.g., the introduction of a C–C bond through cross-coupling or the introduction of a different substituent/functional group through nucleophilic substitution of the Br (Scheme 3).

**Scheme 3:**
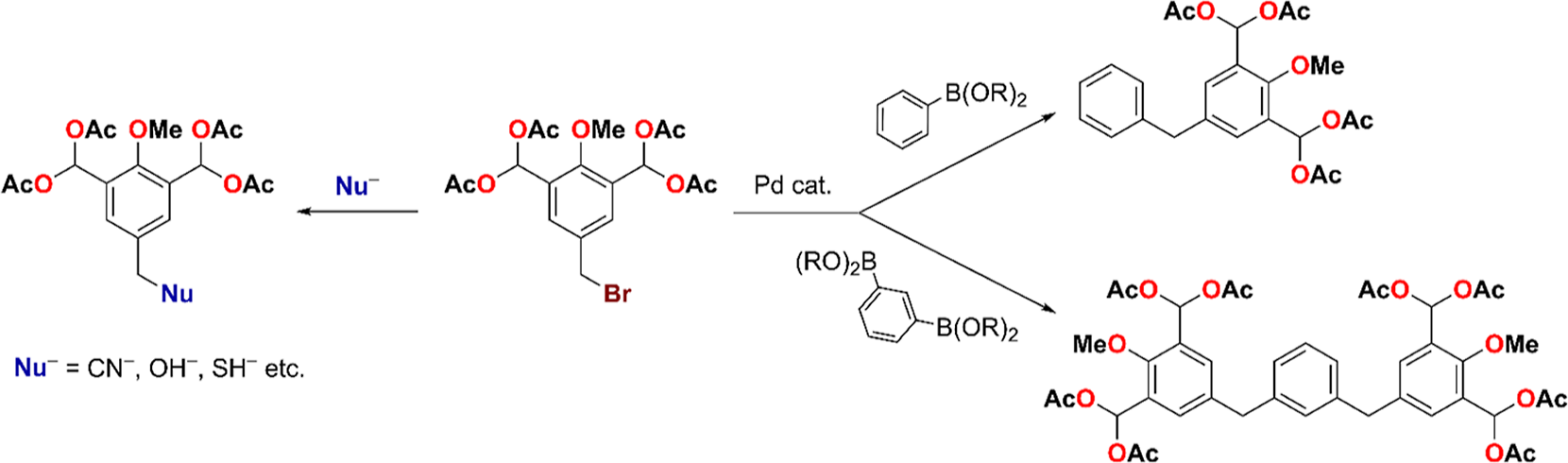
Possible Examples of Further Modifications of Compound **4**

The protecting groups can be removed by first cleaving the methyl ether with BBr_3_,^34^ then followed by acylal deprotection with either acid-catalyzed hydrolysis or sodium hydroxide/potassium carbonate in aqueous THF.^35^ Depending on the specific synthetic goals and targets, the protecting groups could also be removed at any later stage.

## Conclusion

In conclusion, we developed a straightforward, reproducible, scalable, and reliable three-step procedure for the bromination of the benzylic position in 2-hydroxy-5-methylisophthalaldehyde, which has not been previously reported in the literature. The overall yield across the three steps is around 34%. The introduction of benzyl bromide offers a possibility for further functionalization and derivatization of the parent compound for various potential applications.

## Supplementary Material



## Data Availability

The data underlying this study are available in the published article and its Supporting Information.

## Notes

Experimental procedures and NMR and IR spectral copies (PDF)

## †

Department of Chemistry, University of Latvia, Jelgavas iela 1, LV-1004 Riga
