# Supplementary material for: Convenient Benzylic Bromination of 2‑Hydroxy-5-methylisophthalaldehyde
Source: ACS Omega. 2026 Jan 23;11(5):8442–5. doi: 10.1021/acsomega.5c11342 (PMC12903034; doi:10.1021/acsomega.5c11342)
Supplement: Supplementary file 1 [file ao5c11342_si_001.pdf]

Supporting information

Convenient benzylic bromination of 2-hydroxy-5-methylisophthalaldehyde

Nikita Žoglo,[a] Anton Mastitski,[a]* Vladislav Ivanistsev,[b] Nadežda Kongi[a]*

[a]*Institute of Chemistry, University of Tartu, Ravila 14a, 50411 Tartu, Estonia*
[b] *Department of Chemistry, University of Latvia, Jelgavas iela 1, LV-1004 Riga, Latvia*

***Corresponding authors; E-Mail:** anton.mastitski@ut.ee, nadezda.kongi@ut.ee

Contents

General considerations 2
Reaction procedures 3
^{1}H and ^{13}C NMR spectra 5
IR spectra 8
References 10

General considerations

Melting points were obtained on a Stuart SMP10 capillary apparatus and are uncorrected. Nuclear magnetic resonance (NMR) spectra were measured on a Bruker instrument (700 MHz for proton spectra and 176 MHz for carbon spectra), using $CDCl_3$ as a solvent and solvent residual signal as the internal reference for all the measurements. Infrared (IR) spectra were taken using a KBr disc (containing approx. 1–2 weight% of sample) transmittance measuring technique on a Perkin–Elmer Spectrum BX spectrometer. The progress of the reactions was monitored by thin-layer chromatography (TLC) using silica gel 60 F_{254} plates (Merck). Specific eluents are given in the compound characterisation sections. All the yields are based on the masses of the starting reagents. The starting isophthalaldehyde was purchased from BLDPharm. Elemental analysis measurements were carried out using a Vario MACRO CHNS Cube Elemental Analyzer system.

List of abbreviations: Bz – benzoyl; Me – methyl; Ac – acetyl, Boc – tert-butyloxycarbonyl; DIPEA – N,N-diisopropylethylamine; DMF – N,N-dimethylformamide; DCM – dichloromethane; EtOAc - ethyl acetate; Hex – hexane; IR – infrared; MsOH – methanesulfonic acid; NBS – N-bromosuccinimide; AIBN – Azobisisobutyronitrile; NMR – nuclear magnetic resonance; Ph – Phenyl; R_f – retention factor; TLC – thin layer chromatography.

Reaction procedures

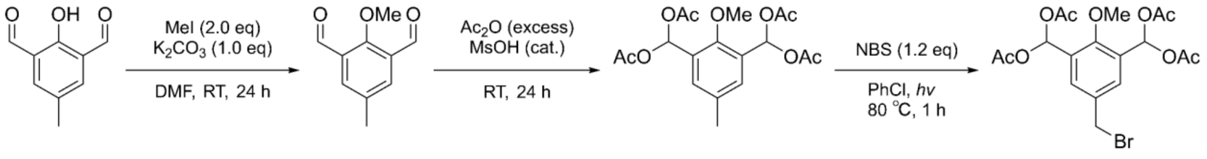

Figure S1. Three-step synthesis of (5-(bromomethyl)-2-methoxy-1,3-phenylene)bis(methanetriyl) tetraacetate (**4**).

Synthesis of 2-methoxy-5-methylisophthalaldehyde (**2**):
A 50 mL round-bottom flask was charged with 2-hydroxy-5-methylisophthalaldehyde (**1**) (1.0 g, 6.10 mmol, 1.0 eq), to which K_2CO_3 (0.85 g, 6.14 mmol, 1.0 eq) and MeI (2.0 g, 14.00 mmol, 2.3 eq) were transferred using DMF (10 mL). The slurry was set to stir overnight at room temperature. The reaction progress was monitored by TLC on the next day, eluting with DCM (R_f (**2**) = 0.2). The TLC probe was prepared by adding two drops of the reaction mixture to 0.1 M $KHSO_4$ (ca. 0.5 mL), followed by EtOAc (ca. 20 drops). Upon completion, the flask's contents were poured into cold H_2O (500 mL) and the yellow powder of compound **2** was separated by filtration and allowed to air-dry. Then, saturated Na_2CO_3 solution (20 mL) was added to the aqueous phase, which was then extracted with EtOAc (4×50 mL). Combined organic fractions were washed with brine (50 mL) and dried over anhydrous Na_2SO_4. Solvent was removed under reduced pressure (ca. 45 ℃) and combined with the precipitate obtained from filtration. Drying the combined solids under vacuum overnight afforded compound **2** (0.97 g, 89%) as a yellow solid. The obtained compound was pure enough to be used as is in the next step.
Mp. 96 – 98 ℃, lit.[1] 91.5 – 92.5 ℃, R_f (DCM) = 0.20. IR (cm^{-1}): 3050, 2960, 2926, 2861, 2756, 1682, 1582, 1476, 1400, 1234, 1145, 1132, 990, 939, 886, 715, 659, 552. ^1H NMR (700 MHz, $CDCl_3$): δ 10.35 (s, 2H), 7.87 (s, 2H), 4.02 (s, 3H), 2.38 (s, 3H); $^{13}C\{^1H\}$ NMR (176 MHz, $CDCl_3$): δ 188.7, 163.7, 135.5, 135.1, 129.8, 66.9, 20.7.

Synthesis of (2-methoxy-5-methyl-1,3-phenylene)bis(methanetriyl) tetraacetate (**3**):
Compound **2** (0.97 g, 5.43 mmol, 1.0 eq) was dissolved in Ac_2O (10 mL) in a 25 mL round-bottom flask, followed by the addition of MsOH (25 µL). The reaction mixture was set to stir overnight at room temperature. The reaction progress was monitored by TLC on the next day, eluting with DCM/EtOAc 20:1 (R_f (**3**) = 0.39). The TLC probe was prepared by adding one drop of the reaction mixture into a saturated solution of $NaHCO_3$ (ca. 0.5 mL), followed by EtOAc (ca. 15 drops). Upon consumption of compound **2**, the cloudy yellow solution was poured into H_2O (25 mL) and then extracted with EtOAc (3×25 mL). Combined organic fractions were washed with brine (15 mL) and dried over Na_2SO_4. Solvent was removed under reduced pressure (ca. 45 ℃), and the product was dried under vacuum overnight. Compound **3** (2.02 g, 97%) was obtained as a crystalline whitish-yellow precipitate. The obtained compound was pure enough to be used as is in the next step.
Mp. 147 – 149 ℃, R_f (DCM/EtOAc 20:1) = 0.39. IR (cm^{-1}): 3003, 2957, 2943, 2844, 1765, 1745, 1489, 1428, 1376, 1250, 1232, 1206, 1088, 1020, 1006, 975, 932, 894, 701, 657, 605, 570. ^1H NMR (700 MHz, $CDCl_3$): δ 7.89 (s, 2H), 7.37 (s, 2H), 3.87 (s, 3H), 2.34 (s, 3H), 2.06 (s, 12H); $^{13}C\{^1H\}$ NMR (176 MHz, $CDCl_3$): δ 168.4, 153.6, 134.5, 129.7, 129.3, 85.3, 63.5, 20.9, 20.7.

Anal. Calcd for $C_{18}H_{22}O_9$: C, 56.54; H, 5.80. Found: C, 56.71; H, 5.41.

Synthesis of (5-(bromomethyl)-2-methoxy-1,3-phenylene)bis(methanetriyl) tetraacetate (**4**): Compound **3** (1.01 g, 2.65 mmol, 1.0 eq) was dissolved in PhCl (10 mL) in a 25 mL round-bottom flask, put in a small silicon oil bath (***NB! Silicon oil is clear and inert to subsequent irradiation***) and brought to 80 ℃ by heating under a 400 W floodlight, while the side of the setup (flask, oil bath, reflux condenser) unexposed to the light was covered in aluminium foil to aid with irradiation and heating. Once the temperature reached 80 ℃, NBS (579 mg, 3.25 mmol, 1.23 eq) was added all at once, and the reaction was stirred for 1 hour. Upon completion, the reaction mixture was allowed to cool to room temperature and then diluted with DCM (50 mL). It was subsequently washed with saturated $NaHCO_3$ (3×20 mL), brine (20 mL), and dried over Na_2SO_4. Solvent was removed under reduced pressure (ca. 45 ℃) and dried under vacuum. The crude product (1.254 g) was dissolved in boiling EtOAc (5 mL), to which hexane (5 mL) was added in small portions until the solution became slightly cloudy. At this point, the heating was stopped, and the solution was allowed to cool to room temperature before being left to stand in the fridge overnight. The mother liquor was pipetted off, crystals were washed with EtOAc/Hex 1:2 (2×3 mL) and then dried under vacuum. The monobrominated compound **4** (572 mg, 47%) was obtained as white/colourless crystals. The compound was pure enough for the majority of synthetic applications, but an analogous second recrystallisation was performed using EtOAc (1.5 mL) and hexane (1 mL). After standing in the fridge overnight, the mother liquor was pipetted off, crystals were washed with EtOAc/Hex 1:2 (2×1 mL) and then dried under vacuum. Finally, the monobrominated compound **4** (477 mg, 39%) was obtained as a white crystalline powder. Mp. 170 – 172 ℃, R_f (DCM/EtOAc 20:1) = 0.50. IR (cm^{-1}): 2989, 2948, 2842, 1752, 1480, 1433, 1377, 1296, 1238, 1200, 1091, 1007, 937, 920, 899, 796, 671. ^1H NMR (700 MHz, $CDCl_3$): δ 7.93 (s, 2H), 7.62 (s, 2H), 4.49 (s, 2H), 3.93 (s, 3H), 2.11 (s, 12H); $^{13}C\{^1H\}$ NMR (176 MHz, $CDCl_3$): δ 168.5, 155.9, 134.5, 130.5, 130.1, 85.1, 63.8, 32.3, 20.9. *Anal.* Calcd for $C_{18}H_{21}BrO_9$: C, 46.87; H, 4.59. Found: C, 46.79; H, 4.25.

^{1}H and ^{13}C NMR spectra

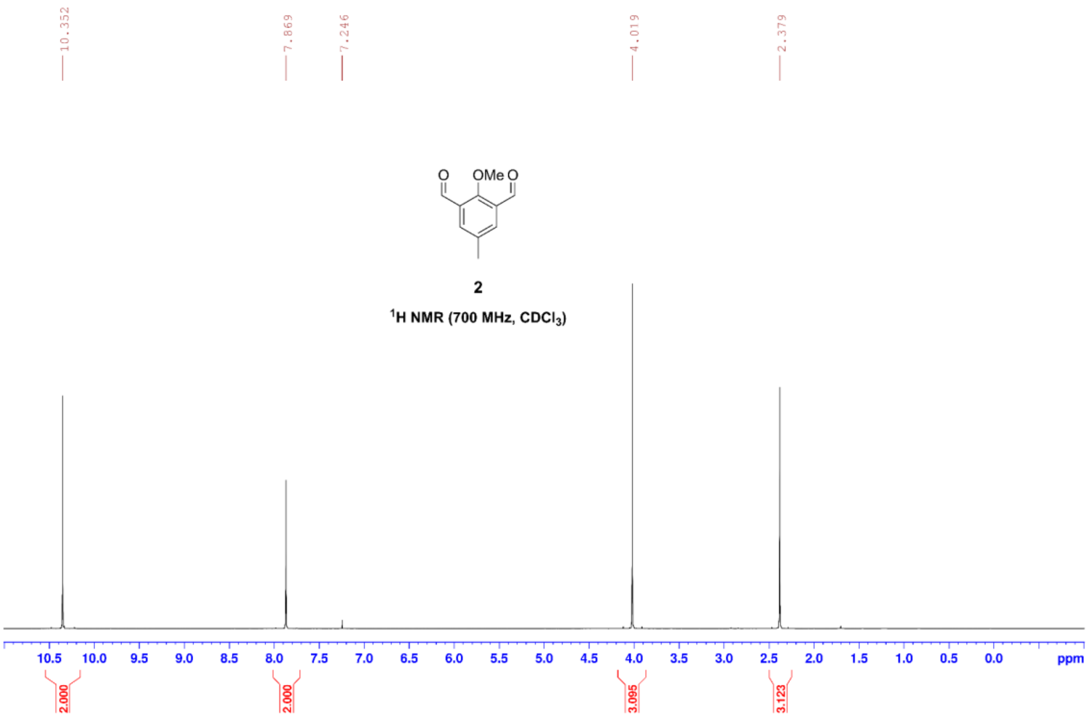

Figure S2. ^{1}H NMR spectrum of compound **2**.

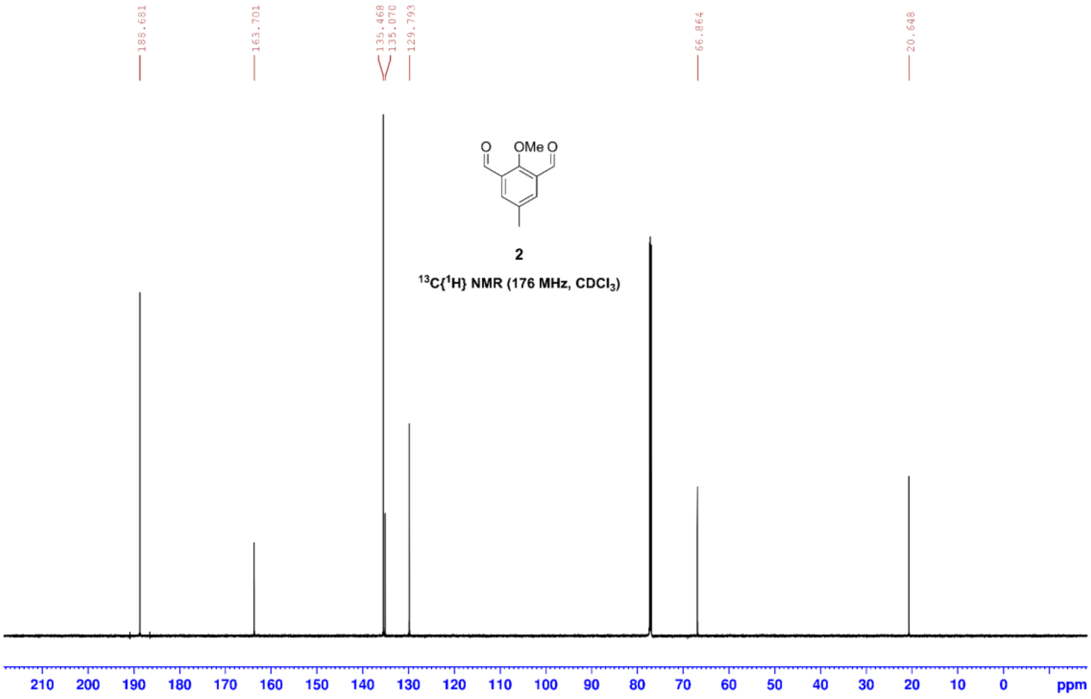

Figure S3. ^{13}C NMR spectrum of compound **2**.

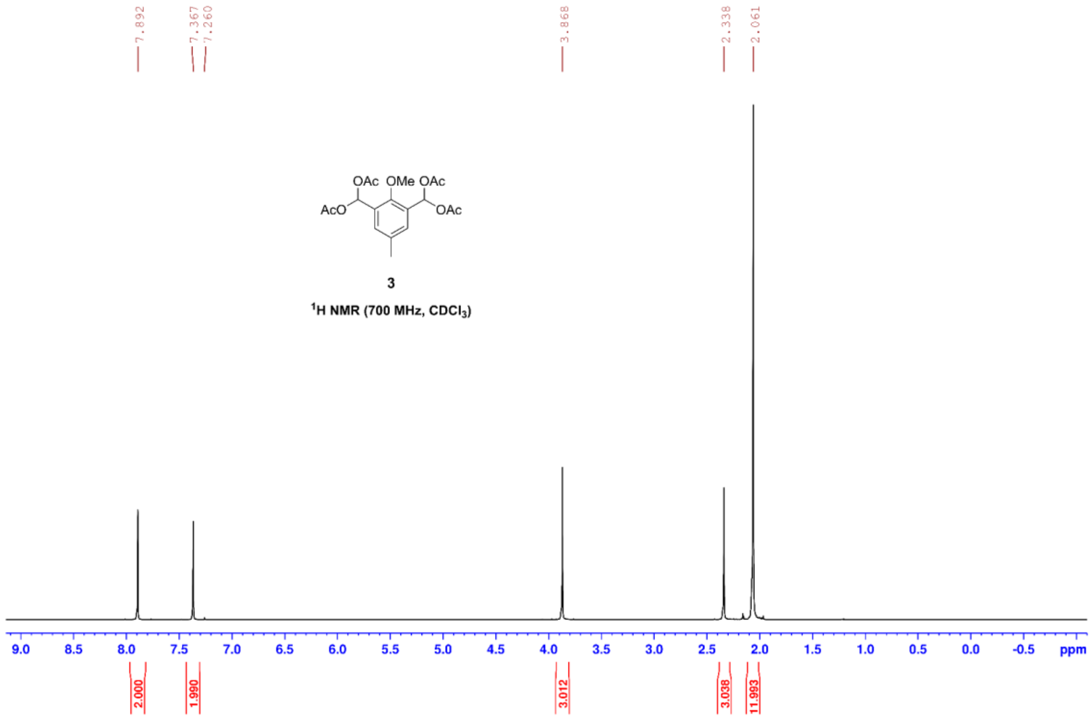

Figure S4. ^{1}H NMR spectrum of compound **3**.

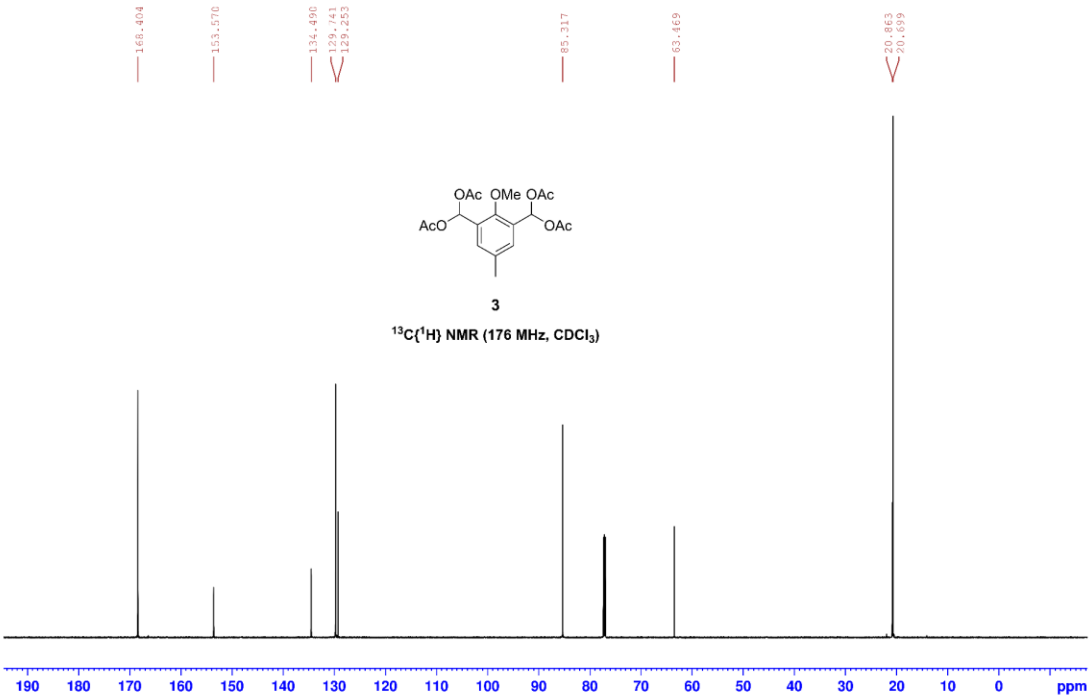

Figure S5. ^{13}C NMR spectrum of compound **3**.

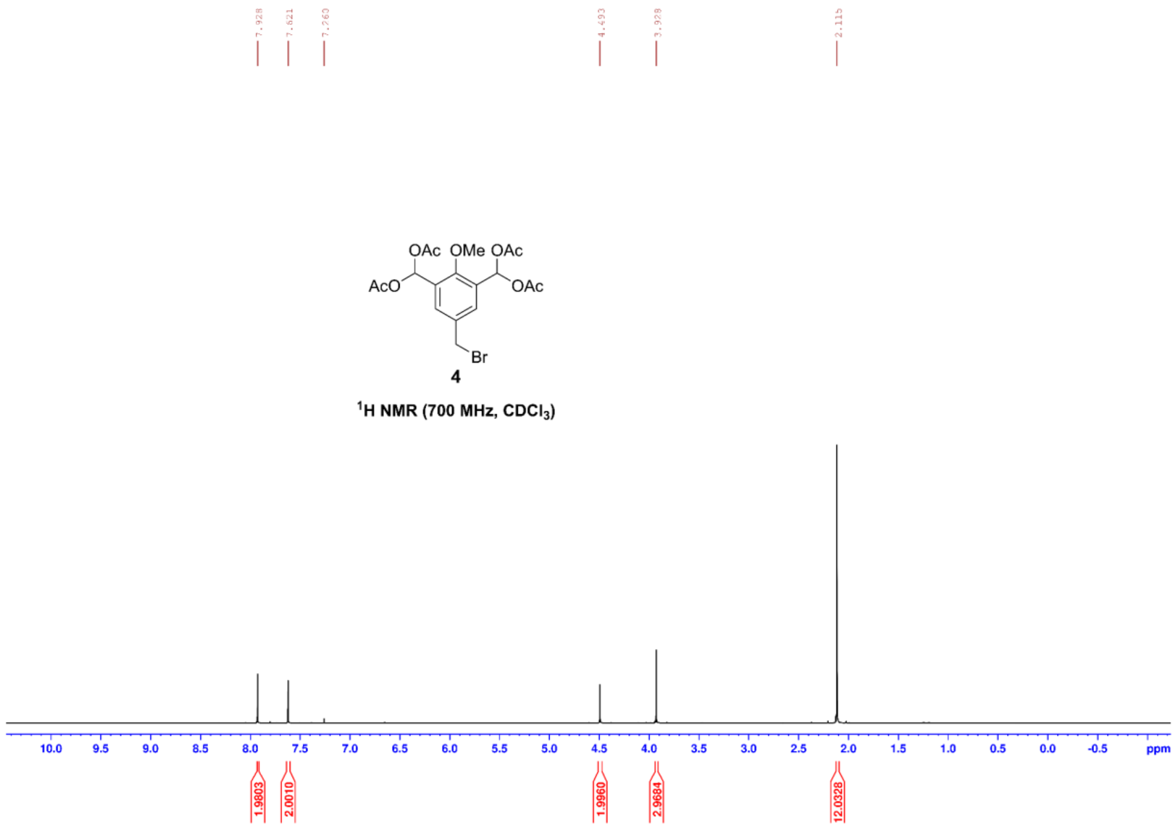

Figure S6. ^{1}H NMR spectrum of compound **4**.

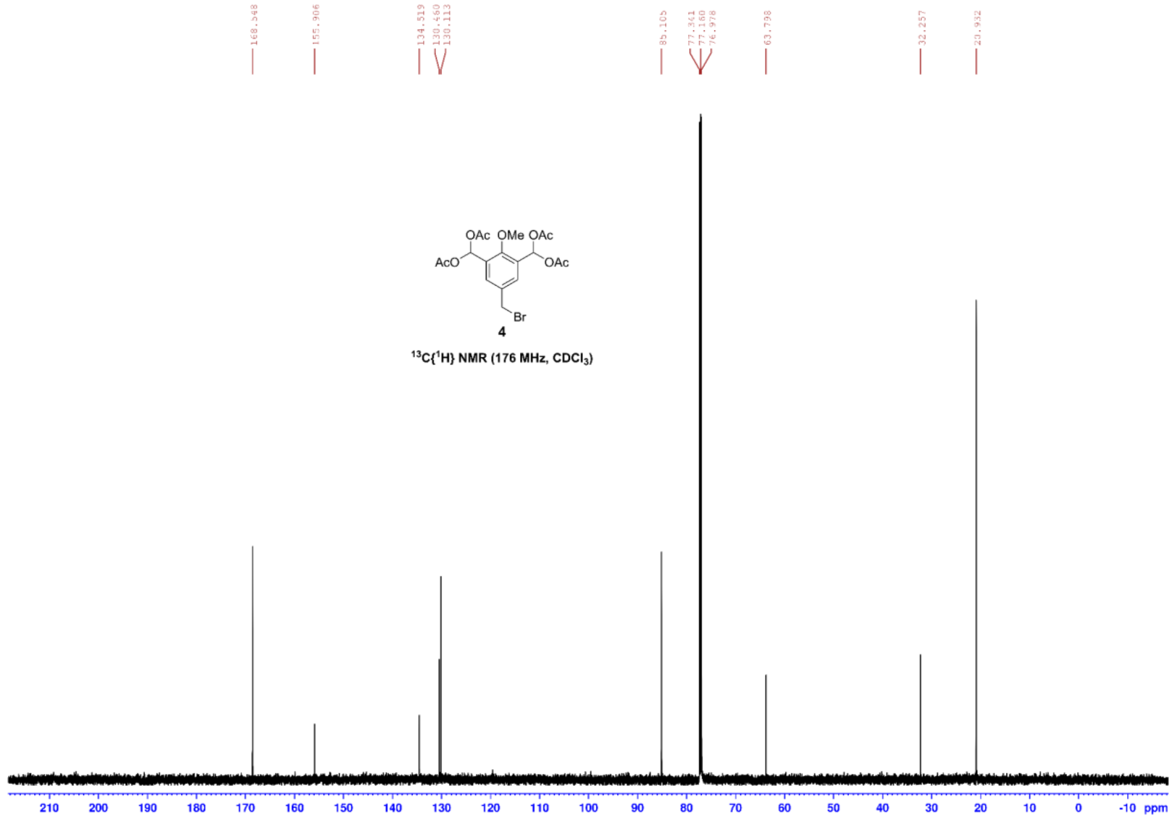

Figure S7. ^{13}C NMR spectrum of compound **4**.

IR spectra

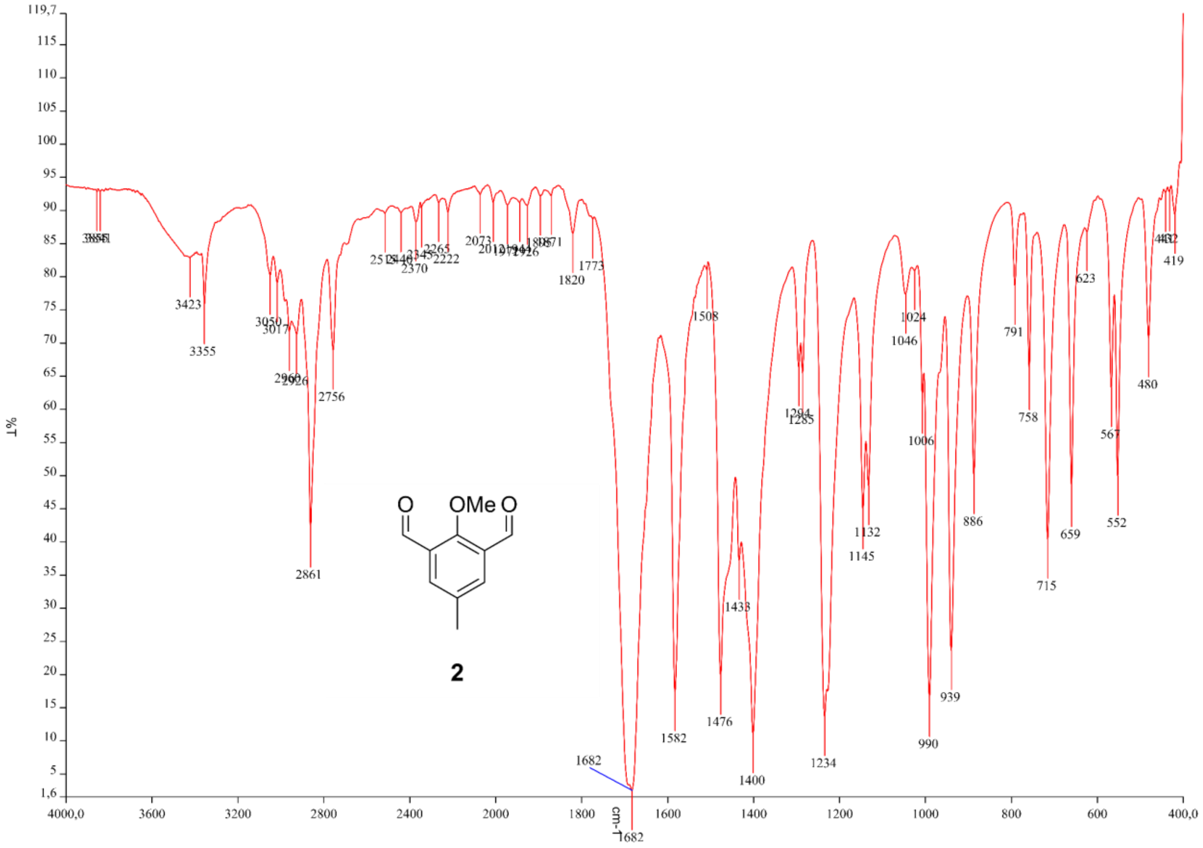

Figure S8. IR spectrum of compound **2**.

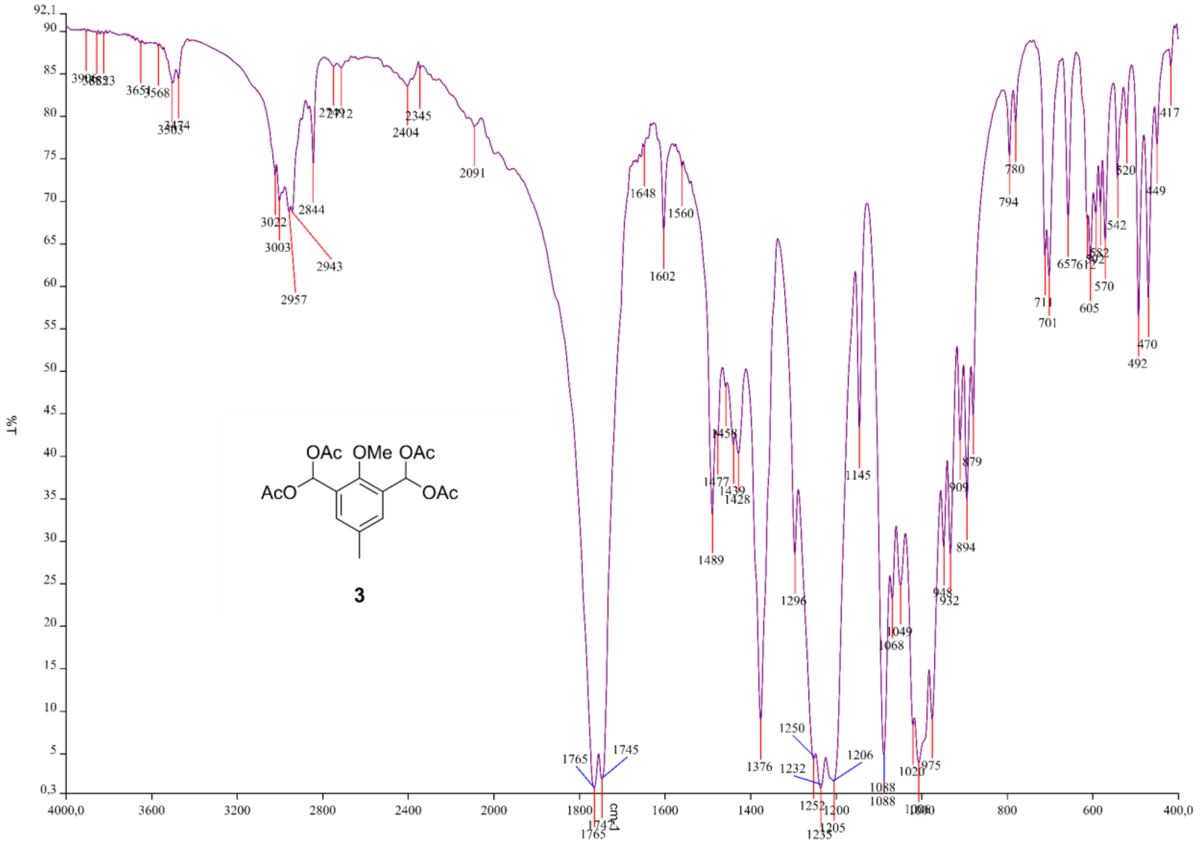

Figure S9. IR spectrum of compound **3**.

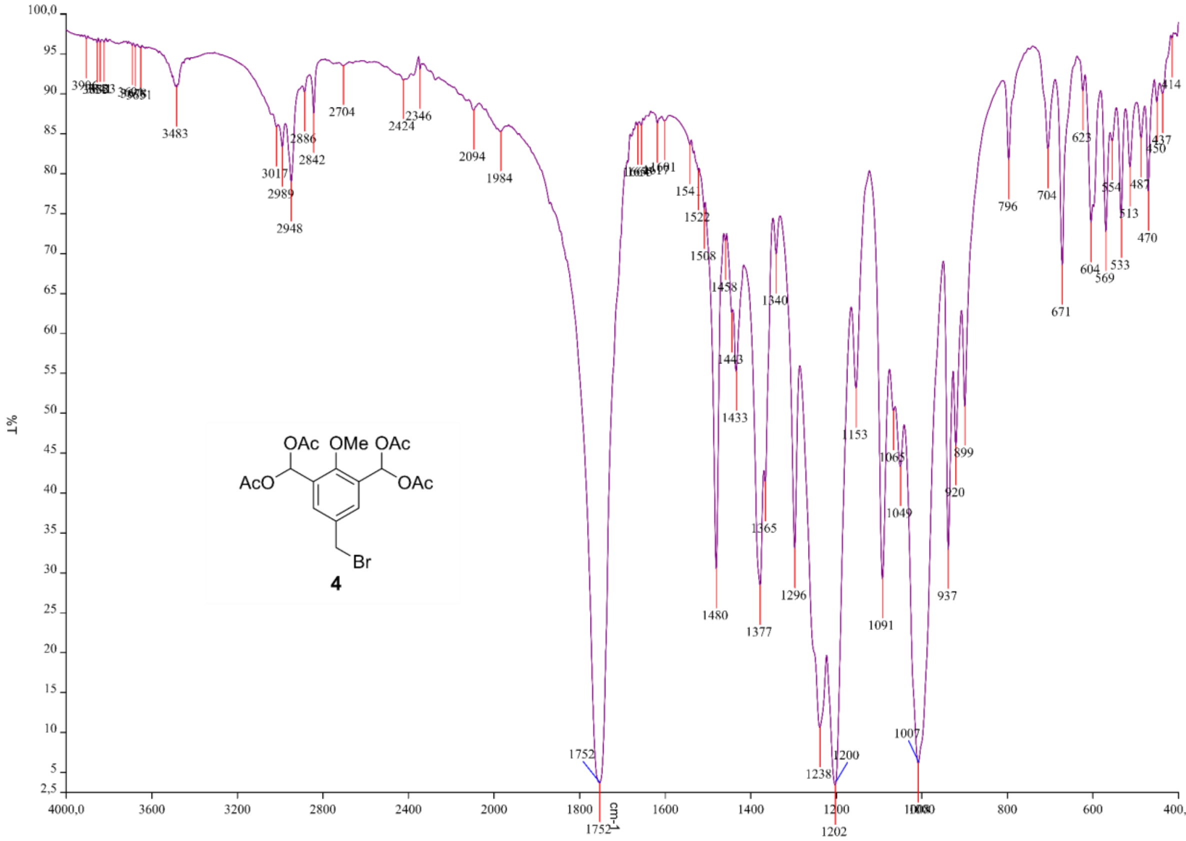

Figure S10. IR spectrum of compound **4**.

References

(1) Morgan, B.; Dolphin, D. Synthesis of Hydrocarbon-Strapped Porphyrins Containing Quinone and Phenolic Groups. *J. Org. Chem.* **1987**, *52* (24), 5364–5374. https://doi.org/10.1021/jo00233a011.
